# Gene expression of Interleukin-18 in rheumatoid arthritis patients on disease modifying anti-rheumatic drug therapy

**DOI:** 10.12669/pjms.35.3.1070

**Published:** 2019

**Authors:** Sabeen Khalid, Muhammad Javad Yousaf, Amir Rashid, Saleem Ahmad Khan

**Affiliations:** 1 *Dr. Sabeen Khalid, MBBS, MPhil. Department of Biochemistry, Aziz Fatimah Medical & Dental College, Faisalabad, Pakistan*; 2 *Muhammad Javad Yousaf, MBBS, FCPS, MHPE. Department of Biochemistry & Molecular Biology, Army Medical College, Rawalpindi, Pakistani*; 3 *Amir Rashid, MBBS, PhD. Department of Biochemistry & Molecular Biology, Army Medical College, Rawalpindi, Pakistani*; 4 *Saleem Ahmad khan, MBBS, FCPS, PhD. Department of Pathology, Army Medical College, Rawalpindi, Pakistani*

**Keywords:** Rheumatoid arthritis, Interleukin-18, Disease modifying anti-rheumatic drugs, Gene expression

## Abstract

**Background & Objectives:**

The hallmark of rheumatoid arthritis is the inflammation that is mediated by the macrophages and monocytes that cause release of pro-inflammatory cytokines like interleukin-18. It is highly expressed in serum of patients suffering from rheumatoid arthritis and has a positive association with disease activity. The aim of this study was to analyze the gene expression of interleukin-18 in rheumatoid arthritis patients on disease modifying anti-rheumatic drug therapy.

**Methods:**

The cross sectional comparative study is conducted at Department of Biochemistry and Molecular Biology and Center for Research in Experimental and Applied Medicine (CREAM-1Lab), Army Medical College, Rawalpindi, in collaboration with Rheumatology Department, Military Hospital, Rawalpindi. Study was conducted on two groups consisting of Group-I of rheumatoid arthritis patients on diseases modifying anti-rheumatic drugs and control Group-II comprising of normal healthy individuals. Non-probability purposive sampling was done from patients and controls. The duration of study was one year i-e from November 2015 to November 2016. Relative quantification of gene expression of interleukin-18 was done by Real time PCR using ∆∆CT method.

**Results:**

Expression analysis for interleukin-18 showed down regulation of gene in rheumatoid arthritis patients as compared to controls.

**Conclusion:**

Interleukin-18 gene shows down regulation in rheumatoid arthritis patients on disease modifying anti-rheumatic drugs therapy.

## INTRODUCTION

Rheumatoid Arthritis (RA) is a prevailing and debilitating autoimmune disease; characterized by chronic inflammation and joint destruction.^1^ It is a systemic disease that causes both cartilage and joint destruction leading to loss of function. It is mostly present at an age of 45-50 years and above, with higher prevalence in females than in males.^2^

Patients of RA are now being clinically diagnosed with the help of American College of Rheumatology / European League Against Rheumatism (ACR/EULAR) classification criteria.^1^ This criterion is based upon four variables i-e small joint involvement, at least 6 months disease duration, positive serology for rheumatoid factor (RF) or anti-cyclic citrullinated peptide (anti-CCP) and presence of any one of the acute phase inflammatory marker i-e Erythrocyte Sedimentation Rate (ESR) or C-Reactive Protein (CRP).^1^,^3^

Its worldwide prevalence is almost 1% that although seems small but due to its disability and deformity it has been a major health problem, with more prevalent in European and Asian Ancestry.^4^,^5^ In Pakistan it is also an upcoming leading health risk towards the growing population; affecting 0.142% people in southern side and 0.55% in northern areas of Pakistan.^6^

The current recommended treatment therapy for RA is that of Disease Modifying Anti-Rheumatic Drugs (DMARD)^7^ in order to lessen the underlying inflammation and to improve quality of patients life.^5^ The current recommended DMARD therapy includes both conventional and biological agents. They can be either used alone or in combination with each other. Commonly used conventional DMARDs include methotrexate, leflunomide, hydroxy chloroquine and sulfasalazine.^8^ While biological DMARDs include tumor necrosis factor alpha inhibitors for example infliximab, rituximab, etanercept, adalimumab.^3^

RA is a disease of multifactorial cause with more than 60% affected by the genetic influence.^9^ Almost 30 genes have been found to be associated with RA susceptibility and these account for more than 50% genetic contribution of RA.^10^ Remaining etiology is being participated by various environmental factors like infection, obesity, smoking and other environmental pollutants.^11^ Among the genetic factors, interleukins play a chief role in causing inflammation that leads to progressive joint destruction. Interleukins (ILs) are the group of pro inflammatory cytokines that play a major role in pathogenesis of RA and its disease progression by stimulating the immune response. ILs are produced and acts on their target cell via its receptor. This collaboration activates the cascade of events that lead to alteration in cell behavior. IL-18, a member of family of IL-1,^12^ is an important pro inflammatory cytokine with a potential role in pathogenesis of RA.^13^ IL-18 is diverse cytokine that is raised in various autoimmune and inflammatory metabolic disorders including rheumatoid arthritis. It is primarily secreted by macrophages and dendritic cells.^14^ It is highly expressed in serum, synovial tissue and synovial fluid of patients suffering from RA and has a positive association with disease activity.^15^ IL-18 accelerates the bone destruction in rheumatoid patients by indirectly stimulating the osteoclasts by up regulation of both soluble and membrane bound receptors i-e Receptor Activator of Nuclear Factor κB Ligand (RANKL) by RA synovial derived T cells. IL-18 contributes to pathogenesis of RA by leukocyte extravasation and by up-regulating adhesion cell molecules. It also releases the chemokines by synovial fibroblast cells of RA and directly as lymphocyte, monocyte and neutrophil chemo-actractant.^12^ There is a positive link between RA pathogenesis and IL-18 gene expression.^16^

The aim of our study was to evaluate the expression of IL-18 in RA patients on DMARD therapy compared to expression in healthy controls, in context with local population. This will help by adding to the genetic pool of our population and providing new era to the field of molecular medicine.

## METHODS

Study was formally approved by ethical review committee of Army Medical College and was conducted at Department of Biochemistry and Molecular Biology and CREAM Lab-1, Army Medical College Rawalpindi in collaboration with Rheumatology Department, Military Hospital, Rawalpindi.

This was cross sectional comparative study done over the period of one year i-e from Nov 2015-Nov 2016. Non probability purposive sampling was done. The study comprised of two groups, Group-I contains thirty diagnosed patients of RA on DMARD therapy, while Group-II comprised of ten normal healthy individuals as controls. Patients of other chronic illnesses like malignancy and patients of any other type of arthritis were excluded from study.

Written informed consent was taken from the patients of RA. Diagnosed patients of RA on continued treatment were enrolled from rheumatology OPD. 2 ml of venous blood sample was drawn from patients and put in the EDTA tube and was brought to CREAM lab for molecular analysis. Specific primers were designed using available sequence at NCBI. Following were the designed primers for IL-18: CGCTTCCTCTCGCAACAAAC as forward primer, and ATGGTCCGGGGTGCATTATC as reverse primer. Total RNA was extracted using Thermoscientific kit, following its standard protocols. Purified RNA was stored at −80 C. cDNA was synthesized using Revert Aid Premium First Strand c DNA Kit. The resulting product was stored at −20 C for further use. Primers were first reconstituted using PCR water. They were optimized on conventional PCR and annealing temperature observed at 56.9°C. Further gel electrophoresis was done on 2 % agarose gel. Quantitative PCR was performed on Cephid Time Smart Cycler. The syber Green q PCR Master Mix universal kit (Thermoscientific) was used for PCR analysis. Smart Cycler was set according to kit´s recommendations and program was started for 40 cycles. IL-18 gene expression was quantified using ∆CT method. ∆∆CT was calculated by following equation

∆CT (test sample) - ∆CT (calibrator sample) = ∆∆CT

Data was analyzed on SPSS 22 version. Numerical variables were expressed as mean ± standard deviation. Qualitative data was expressed as frequency and percentages.

## RESULTS

This cross sectional comparative study comprised of two groups. Group-I consist of thirty patients suffering from RA on DMARD therapy, while the Group-II contains ten healthy individuals as controls. Mean age of Group-I of RA patients was 49.69 ±11.5 and that of control Group-II was 47.4 ± 10.4 years. Among the study population 14 (35 %) individuals were males and 26 (65 %) were females. In patient category, there were 8 (27 %) males and 22 (73 %) females. In control group 5 (50 %) males and 5 (50 %) females were present.

Relative quantification of gene expression was done by ∆∆CT method.^17^ Mean CT values of IL-18 and GAPDH (house-keeping gene) were calculated in RA patients. Mean CT values of IL-18 and GAPDH were also calculated for control group. ∆CT was estimated by measuring the difference between mean CT of GAPDH and IL-18 in both patients and controls. ∆∆CT was calculated by the formula given below:

∆∆CT = ∆CT (patients) -∆CT (controls)

∆∆CT is put into formula 2^-(∆∆CT)^ to measure the fold increase and decrease in gene expression (Table-I). The results by above mentioned formula showed down regulation of IL-18 in RA patients on DMARD therapy in this study population.

**Table-I:** Relative quantification of gene expression of IL-18 by ∆∆CT method.

Group category	Mean CT IL-18	Mean CT GAPDH	∆CT	∆∆CT
Patients	30.01	23.63	6.38	1.58
Controls	28.88	24.08	4.80	

## DISCUSSION

RA is an auto immune disease of chronic inflammatory nature associated with joint destruction and deformity leading to loss of function. It is a multifactorial systemic disease with symmetrical distribution affecting the synovial joints.^18^ More than 50% RA is being contributed by genetic influence.^19^

The aim of this study was to find the significance of gene expression of IL-18 in RA patients in context with our local population, so that approach towards the treatment of patient should be scrutinized. IL-18 expression has been recognized in monocyte-macrophage system, osteoblasts, and chondrocytes and recently in Fibroblast-Like Synoviocytes (FLS).^20^ IL-18 is a member of IL-1 family, which mediates it´s pro-inflammatory effect and cartilage destruction by T-cell activation.^21^

Our study showed down regulation of IL-18 gene in RA patients when compared with controls, quantified by RT-PCR by peripheral blood samples. These results are in correspondence with the previous study conducted in Germany by Moller and his co-researchers in 2001.^20^ It showed that constitutive expression of IL-18 is significantly reduced in Peripheral Blood Mononuclear Cells (PBMC) in patients suffering from RA.

A study conducted in Ireland also showed significant down regulation of IL-18 expression after response to the standard treatment for RA.^22^ This study also measured the serum levels of IL-18 by ELISA, which also showed marked reduction in serum levels of IL-18 in RA patients when given the standard methotrexate treatment for reducing the crippling and debilitating effect of inflammation.

Study is also available where initially there was increased expression of IL-18. Later on when switched to newer and better DMARDs the expression of IL-18 gene showed relative down expression as compared to earlier response of patient toward treatment.^4^

IL-18 mRNA is also being detected in synovial fluid and synovial membrane of RA patients as well. But studies have shown that IL-18 levels are significantly raised in synovial tissues as compared with serum of RA patients.^23^

Edward and it´s co-researchers in USA also detected the mRNA expression of IL-18 in peripheral blood sample of RA patients.^4^ Their results showed the up-regulation of IL-18 mRNA expression in patients suffering from RA that are not responsive to DMARD treatment as compared to those that responded well to therapy.

X.-T. Shao and L. Feng also reported the increase gene expression of IL-18 and it´s receptor in RA patients as compared to osteoarthritis patients considered as control group. This increase is being reported in both synovial tissue, synovial fluid and in peripheral blood sample as well. This expression of IL-18 and IL-18BP were estimated using Real time PCR and Western Blot.^24^

Another study conducted also showed up regulation of IL-18 expression in RA patients when compared with control Group-In peripheral blood samples. Normal healthy individuals were taken as control population. This showed the highly active disease in patient population. IL-18 is significantly raised in synovial tissue, synovial fluid and patient′s serum as well.^25^

Limitations of this study: Expression of IL-18 with individual DMARDs cannot be analyzed because of time limitations and lack of enough finances. Similarly any relationship of iL-18 expression and response to therapy could not be ascertained which are some of the limitations of this study. However, this study is of significance in context with the local population and adds to the genetic pool of population.

## CONCLUSION

The study showed down regulation of IL-18 in RA patients responsive to DMARD therapy.
